# On the Origin of the Anomalous Behavior of Lipid Membrane Properties in the Vicinity of the Chain-Melting Phase Transition

**DOI:** 10.1038/s41598-020-62577-9

**Published:** 2020-04-01

**Authors:** Alexander Kuklin, Dmitrii Zabelskii, Ivan Gordeliy, José Teixeira, Annie Brûlet, Vladimir Chupin, Vadim Cherezov, Valentin Gordeliy

**Affiliations:** 1 Moscow Institute of Physics and Technology, Dolgoprudny, Russian Federation; 2 Joint Institute for Nuclear Research, Dubna, Russian Federation; 3 Institute of Biological Information Processing (IBI-7: Structural Biochemistry), Research Centre Jülich, Jülich, 52425 Germany; 4 Georgetown University, Washington, USA; 5 Laboratoire Leon Brillouin (CEA/CNRS), CEA Saclay, 91191 Gif-sur-Yvette, France; 6 Department of Chemistry, Bridge Institute, University of Southern California, Los Angeles, CA 90089 USA; 7 Institute de Biologie Structurale Jean-Pierre Ebel, Université Grenoble Alpes-Commissariat à l’Energie Atomique et aux Energies Alternatives-CNRS, Grenoble, France

**Keywords:** Biological physics, Chemical physics

## Abstract

Biomembranes are key objects of numerous studies in biology and biophysics of great importance to medicine. A few nanometers thin quasi two-dimensional liquid crystalline membranes with bending rigidity of a few kT exhibit unusual properties and they are the focus of theoretical and experimental physics. The first order chain-melting phase transition of lipid membranes is observed to be accompanied by a pseudocritical behavior of membrane physical-chemical properties. However, the investigation of the nature of the anomalous swelling of a stack of lipid membranes in the vicinity of the transition by different groups led to conflicting conclusions about the level of critical density fluctuations and their impact on the membrane softening. Correspondingly, conclusions about the contribution of Helfrich’s undulations to the effect of swelling were different. In our work we present a comprehensive complementary neutron small-angle and spin-echo study directly showing the presence of significant critical fluctuations in the vicinity of the transition which induce membrane softening. However, contrary to the existing paradigm, we demonstrate that the increased undulation forces cannot explain the anomalous swelling. We suggest that the observed effect is instead determined by the dominating increase of short-range entropic repulsion.

## Introduction

Fluid-lipid bilayers are essential components of cell membranes and ubiquitous structural elements of living matter^1,2^, such as the regulation of protein functional activity by lipid bilayer structure, propagation of signals in neurons, and membrane dynamics^3–5^. Phospholipids dispersed in water, as well as some biological membranes, such as, for example, myelin membranes of neurons^6^, usually self-assemble into multilamellar structures (MLVs) that can be described as stacks of two-dimensional smectic liquid crystals. Stacking of lipid bilayers involves three types of intermembrane interactions: Van der Waals attraction, short-range repulsion, which decays exponentially, and a long-range entropic repulsion due to thermal undulations of lipid bilayers^7,8^. At short distances, between lipid membranes (*d*_*w*_ < 20 Å), the interbilayer repulsion is dominated by the short-range repulsion. However, at longer distances, Helfrich repulsion forces may be comparable with Van der Waals attraction^9^. The balance between these forces is also influenced by short-ranged repulsive forces whose origin is still under discussion^10–12^. An important characteristic of lipid bilayers is their phase transition from a gel phase to a liquid-crystalline phase (the main phase transition)^13–15^. The theories and models of phospholipid phase transitions have previously been reviewed in great detail in a number of early papers^16–18^ and reviews^19–23^. Available experimental data justify that the transition has both first- and second-order features^24^. Sharp charges, characteristic of the first order phase transition, are observed in various physical properties, such as specific heat^25^, density^26^, intramolecular vibration^27^, orientational order of hydrocarbon chains^26,28^, intramolecular and intermembrane distances^29^, lateral diffusion coefficient^30^, and partitioning of a nitroxide-spin label^31^. These abrupt changes in physical properties are due to the isothermal first-order transition. On the other hand, a broad anomalous peak or dip occurs near the transition temperature in ultrasonic velocity and absorption^32,33^, permeability^34,35^, relaxation times in various time ranges^36,37^, and two-dimensional compressibility^38^. This kind of anomaly is usually observed when structural fluctuations are enhanced as in the second-order phase transitions. Therefore, the gel-to-liquid crystal transition of the lipid bilayer is considered a weak first-order transition that shows latent heat as well as pseudocritical anomalies. To interpret correctly the results of experimental studies of lipid bilayer properties in the vicinity of the transition temperatures it is essential to take into account that for DPPC membranes the temperatures corresponding to the isothermal first-order transition and the anomaly (a dip) usually associated with structural fluctuation differ by 0.6 °C, while the width of the dip is about 0.3 °C.

The first attempts to explain the origin of the anomalous effect happened in 1994 where the group of O. Mouritsen directly observed deviation in the repeat distance of unilamellar vesicles (ULVs) using x-ray diffraction and small-angle scattering techniques^39^. In this work they experienced significant problems with experimental data interpretation and model limitations. X-ray diffraction methods give an insight of a bilayer structure, although experimental data also account for the stacking of the bilayers, that is not non-trivial to decouple^23,40^. Also existing theories, that describe smectic liquid crystals scattering require significant corrections in the critical region, where lipid bilayers are strongly disturbed^41^. The anomalous swelling effect was hypothesized to be explained by an increase in the intermembrane distance induced by the increased Helfrich repulsion forces and accompanied by membrane softening^39,41,42^. However, proposed theory relies on a phenomenological parameter that couples density fluctuations to bending rigidity, which was not determined experimentally^9^. An alternative explanation that the anomalous swelling is primarily caused by an increase of the bilayer thickness, while density fluctuations are not sufficient to cause the effect^43,44^. Later, the membrane softening in the critical region was confirmed with various experiments, while no evidence was found to support the abnormal increase in the bilayer thickness^45,46^. The studies about the influence of hydration^47^, lipid chain length^48–50^ and osmotic pressure^51,52^ on the anomalous swelling effect, also indirectly support the initially proposed theory^39^, although do not bring a detailed explanation of the effect. Importantly, the anomalous swelling effect is not coupled to the formation of a ripple phase and occur at the spinoidal point T^*^, which is close, but do not coincide with the main phase transition temperature *T*_*m*_^53^. The difference between these two temperature can be estimated as (*T*_*m*_ − T^*^)/*T*_*m*_ ≃ 10^−2^, according to the^42^.

Importantly, the balance of forces strongly depends on the accurate measurement of the intermembrane distance (5), which requires careful decoupling of structure factor and accounting for hydration effects. Here, we present a comprehensive study of the anomalous swelling problem by a complementary use of SANS and neutron spin echo (NSE) techniques. With SANS, we studied the temperature dependences of the repeat distance of MLVs and the lipid bilayer thickness of unilamellar vesicles (ULVs) in excess water. Using these data, we directly observed that the increase in the repeat distance is mainly due to the increase in the intermembrane distance. With NSE experiments, we probed thermal undulations of MLVs and determined the temperature dependence of the bending rigidity in the vicinity of the main transition.

## Results

**Figure 1:**
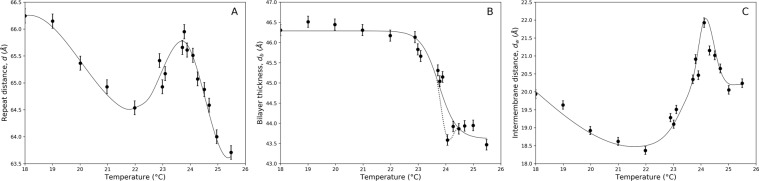
Temperature dependencies of DMPC MLV parameters obtained from SANS data. (**A**) Repeat distance between lipid bilayers obtained with 4% (v/v) DMPC MLVs in *D*_2_*O*. (**B**) Bilayer thickness obtained with 1% (v/v) DMPC ULVs in *D*_2_*O*. Anomalous behavior of the bilayer thickness in the phase transition region is denoted by dashed line. (**C**) Intermembrane distance calculated by subtracting the bilayer thickness from the repeat distance.

**Figure 2:**
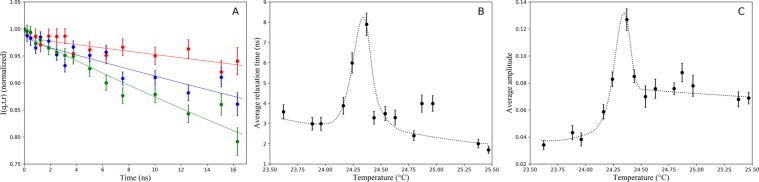
NSE spectra of DMPC ULVs. (**A**) Representative examples of NSE data for 4% (v/v) DMPC ULVs in *D*_2_*O* and their double exponential fits at *T* = 22.16 °C, 23.96 °C and 24.36 °C, colored in red, blue and green, respectively. (**B**) Average relaxation time and (**C**) amplitude of DMPC membrane undulations in the vicinity of the chain-melting phase transition (*T*_*m*_ = 24.3 °C) calculated from a double exponential fit of NSE spectra. Behavior of the parameters is shown by the dotted line.

**Figure 3:**
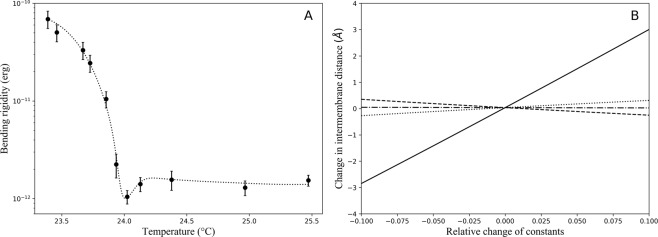
Estimated impact of the key parameters to the anomalous swelling effect. (**A**) Temperature dependence of the lipid bilayer bending rigidity calculated from the NSE spectra. Predicted behavior of the bending rigidity is shown with dotted line. (**B**) Predicted changes in the intermembrane distance induced by variations in the decay length (*λ*), a Hamaker constant (*H*), short range repulsion pre-exponential factor (*P*_0_) and bending rigidity (*K*_*c*_) plotted as solid, dashed, dotted and dash-dotted lines respectively, calculated using Eq. (4).

First, to determine the temperature dependence of the repeat distance we performed SANS studies with 4% (v/v) DMPC MLVs in *D*_2_*O*. Here, the repeat distance *d* was calculated using the Bragg equation in the temperature range of 18–26 °C, after performing normalization, background subtraction and correction for scattering from non-oriented multilayers. Representative examples of the diffraction data are presented in Fig. S1A (see Supplemental Material). The behavior of the repeat distance of DMPC multilayers in this temperature range is shown in Fig. 1A. The anomalous increase of the repeat distance is clearly visible in the vicinity of the phase transition. To avoid calculations of the membrane thickness from the repeat distance, which would invoke previously discussed coupling problem^39,43^, we determined the bilayer thickness *d*_*b*_ directly from SANS using 1% (v/v) DMPC ULVs in *D*_2_*O*. The scattering intensity data were fitted using the following equation:

1 $$I(q)=\frac{{I}_{0}}{{q}^{2}}exp(-{R}_{T}^{2}{q}^{2})$$

where *R*_*T*_ is the bilayer radius of gyration. The bilayer thickness *d*_*b*_ was calculated using the formula ${d}_{b}={R}_{T}\sqrt{12}$, with subsequent correction for inhomogeneity of the membrane using the approach described in^9^. Figure 1B shows the change in the bilayer thickness *d*_*b*_ of DMPC ULVs measured in the temperature range of 18–26 °C. This curve shows that the bilayer thickness monotonously decreases through the transition region, with an additional small anomalous dip occurring in the very narrow region near the phase transition. This anomalous behavior is observed at the same temperature as that for the repeat distance. Therefore, we conclude that these effects are likely related and can be explained by the same phenomenon. The intermembrane distance *d*_*w*_ was calculated for each temperature by subtracting the bilayer thickness *d*_*b*_ from the repeat distance: *d*_*w*_ = *d* − *d*_*b*_ and is shown in Fig. 1C. The data clearly demonstrate that the anomalous swelling is due to the increase in the intermembrane distance and not due to the change in the membrane thickness. Thus, the SANS experiments directly showed that the intermembrane distance anomalously increases in the vicinity of the phase transition. This means that the balance of the intermembrane interactions is changed; however, the SANS experiments alone cannot resolve the question about the nature of this phenomenon. To identify the origin of the anomalous swelling, we studied dynamics of lipid vesicles via NSE technique, which provides information about lipid membrane undulations with appropriate spatial and temporal resolutions. The quasielastic coherent scattering from undulating and simultaneously diffusing vesicles and microemulsion droplets is described in details in^54,55^. In brief, the time- and momentum-dependent scattering intensity *I*(*q*, *t*) can be written as:

2 $$I(q,t,r)={e}^{-D{q}^{2}t}{V}_{s}^{2}{(\Delta \rho )}^{2}{I}_{1}(q,t,r)$$

where *I*_1_ is given by:

3 $${I}_{1}(q,t,r)={f}_{0}(qr)+\sum _{l > 1}{f}_{l}(qr)\left\langle {a}_{l}\right\rangle {e}^{-{w}_{l}t}$$

where *f*_0_(*qr*) is the static form factor term and *f*_*l*_(*qr*) is the *l*_*th*_ quasielastic form factor, responsible for undulations. With temperature increasing from 23 °C to 25 °C, the *D*_2_*O* viscosity is reduced by 5%^56^. As it follows from our SANS data, the radius of vesicles increases by 7% in the same temperature range (the bilayer thickness decreases by 6% and the volume per lipid increases by 4% during the phase transition)^20^. Therefore, for freely diffusing rigid vesicles, the NSE decay time should remain nearly the same before and after the phase transition. To verify whether undulations exhibit an anomalous behavior in the vicinity of the transition, we used a single exponential fit for all the NSE spectra and calculated the decay times in the full temperature range (see Fig. S2 in Supplemental Material). The data demonstrate that the decay time decreases in the vicinity of the phase transition by a factor of 3. Therefore, such decrease in the decay time, is due to an additional contribution to the dynamic scattering corresponding to undulations of lipid membranes. We fitted the NSE experimental spectra by using (2), taking into account both components (diffusion and undulation parts) using only the dominating (*l* = 2) part of the quasielastic term. Representative examples of the experimental data fitted with double exponential fit are shown in Fig. 2A. The obtained average relaxation time and the average amplitudes related to the quasielastic term of (3) are shown in Fig. 2B,C, correspondingly. The anomalous behavior of these parameters in the vicinity of the phase transition is observed at the same temperature as it was for the repeat distance and the lipid bilayer thickness determined by SANS. Thus, the NSE data directly show the effect of membrane softening. At temperatures *T* < 23.5 °C the vesicles are in the gel phase, and therefore can be considered rigid. In this temperature range, we used a single exponential fit for the NSE spectra and determined the average vesicles radius *R*_0_ = 430 Åat *T* = 22.1 °C The NSE spectra at temperatures *T* > 23.5 °C were fitted by (2) taking into account polydispersity. The temperature dependence of the bending rigidity determined from the spectra is shown in Fig. 3A. The data show that the bending rigidity of the ULVs drops considerably in the narrow interval about 0.3 °C around 24.3 °C (the temperature common for all observed anomalies described in this paper). For lipid bilayers in the liquid-crystalline phase (*T* > 25 °C) we obtained *K*_*c*_ = (1.5 ± 0.2) ⋅ 10^−12^*erg* which is in a good agreement with previous results^57,58^, but the bending rigidity further decreases by 33% (*K*_*c*_ ≈ 1.0 ⋅ 10^−12^*erg*) in the vicinity of the main phase transition. However, an important question (which we discuss below) is whether this reduction of the bending rigidity (Helfrich undulations) can account for the anomalous increase in the intermembrane distance. Another important question is whether this change in the membrane elasticity could be explained by the reduction of the membrane thickness observed in the SANS experiments. Our calculations show that, despite the fact that the bending rigidity depends strongly on the membrane thickness (it scales with the thickness nearly quadratically^59^), the reduction of the membrane thickness cannot completely account for the change of the membrane bending elasticity. Thus, critical density fluctuations lead to a more disordered lipid bilayer and therefore to the reduction of the bending rigidity to its softening by a factor of 1.5 as it is observed in the experiment.

One can estimate the effect of the anomalous behavior of the bending rigidity on the intermembrane distance by using the following balance of forces:

4 $${P}_{srr}+{P}_{und}={P}_{VdW}$$

where Van der Waals attraction is:

5 $${P}_{VdW}=\frac{H}{6\pi }\left(\frac{1}{{d}_{w}^{3}}-\frac{2}{{({d}_{w}+{d}_{b})}^{3}}+\frac{1}{{({d}_{w}+2{d}_{b})}^{3}}\right)$$

The short-range repulsion is:

6 $${P}_{srr}={P}_{0}{e}^{-{d}_{w}/\lambda }$$

and the undulation repulsion^9^ is:

7 $${P}_{und}=\frac{3{\pi }^{2}{(kT)}^{2}}{128{K}_{c}{d}_{w}^{3}}$$

For a bilayer under normal conditions, typical values of the decay length of the short-range forces in the liquid-crystalline phase lie between 1.8 Åand 2.2 Å^60^ and the pre-exponential factor is about *P*_0_ = 3 ⋅ 10^10^ *dyn*∕*cm*^2^. Using Eqs. (4–7) one can estimate that the decrease in the bending rigidity from 1.5 ⋅ 10^−12^ *erg* to 1.0 ⋅ 10^−12^ *erg* will barely cause any change in the inter bilayer distance (depending on the value of *λ*, one can achieve at most a 0.1 Åincrease). This is because, in equilibrium, the undulation term is about two orders of magnitude smaller than the Van der Waals and the short-range repulsion terms. Therefore, changing the value of *K*_*c*_ does not substantially affect the force balance. Thus, to explain the increase in the intermembrane distance by almost 2 Å, it is absolutely necessary to have either the Hamaker constant *H* or the pre-exponential factor *P*_0_ or *λ* changed at the critical point, compared to their values in the liquid-crystalline phase. Our analysis shows that the predicted change is most sensitive to the change in *λ* (Fig. 3B). A mere 6% increase in the value of *λ* can ensure that the theoretical estimate matches the measured increase in the intermembrane distance. One needs the Hamaker constant to decrease by about a factor of two at the critical point to explain the observed increase. As for the pre-exponential factor, one would need an almost 80% increase in its value at the critical point to match the change in the interbilayer thickness observed in the experiment.

## Discussion

To summarize, our SANS data obtained with MLVs and ULVs directly showed that the abnormal behavior of the repeat distance between lipid bilayers in the vicinity of the main phase transition is due to the increase in the intermembrane distance. The NSE experiments with ULVs demonstrated that, indeed, as it was suggested by Lemmich *et al*.^41^, the bending rigidity of lipid membranes decreases in the region of the anomalous membrane swelling. This result is also in good agreement with the previous data on ion permeability of the bilayer in this region^35^, since the ion permeability, *p* is proportional to the square of the density fluctuations: $p\propto \left\langle | \delta \rho {| }^{2}\right\rangle$. According to the thermodynamic theory of fluctuations the relative density fluctuation is

8 $$\frac{\left\langle | \delta \rho {| }^{2}\right\rangle }{{\rho }^{2}}=\frac{{k}_{B}T\rho }{K}$$

where *K* is the area compressibility^19,20^. For lipid bilayers the bending rigidity modulus can be related to compressibility as *K*_*c*_ ∝ *Kd*^2^, where *d* is the bilayer thickness^61,62^. Therefore $p\propto {d}^{2}{K}_{c}^{-1}$ and the critical softening of the bilayer directly induce anomalous behavior of the ion permeability. With our data one can estimate $\frac{\delta d}{d}\approx 2.5 \%$ and $\frac{\delta {K}_{c}}{{K}_{c}}\approx 33 \%$ that would give relative increase of ion permeability in the anomalous region for about $\frac{\delta d}{d}\approx 25 \%$, which is in good agreement with previously reported data^34,35^. Furthermore, the experimental data also showed that the decrease in the bending rigidity cannot be explained just by the decrease in the membrane thickness. This means that the state of the lipid bilayer changes anomalously in this region; the membranes are softened, what is naturally explained by critical fluctuations. Thus, the experimental results directly support the existence and significance of critical fluctuations. However, our estimations showed that the increase in undulations does not explain the anomalous swelling of the membrane. Contrary to previous assumptions^15,48^, we found that the undulation term contributes very little to the force balance, thus does not explain the experimentally observed increase in the intermembrane distance. We show the balance of forces is highly sensitive only to the change of *λ*. We argue that a change in the value of the decay length *λ* of the short-range repulsion in the vicinity of the phase transition is the reason of the anomalous increase in the intermembrane distance. It is quite natural to expect this in the framework of the entropic theory of the short-range forces.

We should mention that phenomenological theory by Seifert and Langer^63^ predicts that the relaxation rate of undulating lipid membranes may depend not only on bending rigidity of membranes and viscosity of water but also on the interbilayer friction. It would imply the presence (under specific conditions) of a term in the relaxation rate, scaling with the wave vector *q* as *q*^2^, in addition to the term *Γ*(*q*) ∝ *q*^3^. However, the experiments did not provide a definite proof of this^64^. Moreover, more recent work on NSE studies of lipid membranes (also with lipid vesicles under similar to our conditions^65^) showed that *Γ*(*q*) ∝ *q*^3^ and concluded that "theory of Zilman and Granek for the bending motion of a single membrane^66^ should be applicable here as also shown in similar studies of ULVs dynamics with NSE”^67,68^. Those are the reasons why we used the results of the classical approach described by the theory by Milner and Safran^54^ and Zilman and Granek^66^.

Neutron inelastic scattering experiments support the presence of out-of-plane fluctuations at different time scales in the range of 0.5–3 Å^18^. These data support the significance of collective^7^ and diffusive^10,11^ out-of-plane membrane fluctuations. Therefore, the results indirectly support our results on the significance of both types of motions in the vicinity of the dip. According to the theory^11^, an increase in the amplitude of the out-of-plane fluctuations of lipid molecules leads to an increase in the decay length of the short-range forces *λ* and therefore membrane swelling.

## Methods

### Sample preparation

1,2-dimyristoyl-sn-glycero-3-phosphocholine (DMPC) was obtained from Sigma-Aldrich Co. and used without further purification. MLV and ULV samples were prepared as described in^9^. In brief, MLVs were prepared by dissolving 60 mg lipid in 100 *μl* ethanol. The solvent was subsequently evaporated under a gentle stream of dry nitrogen and further dried under vacuum to remove traces of the solvent. MLVs were formed by hydrating the dry lipid (4% (v/v) lipid/*D*_2_*O* ratio) and subsequent vortexing repeated several times at *T* > *T*_*m*_. ULVs were formed by hydrating the same dry lipid sample (1% (v/v) lipid/*D*_2_*O*) and subsequent extruding repeated several times (more than 20) using a small-volume extrusion apparatus (Avanti). ULVs for NSE experiments were prepared following the same protocol but at 4% (v/v) lipid/*D*_2_*O* in order to increase signal-to-noise ratio.

### Small-angle scattering experiments

SANS experiments were performed using the time-of-flight YuMO (IBR-2, Dubna) instrument^69^. The data were collected on samples in quartz cuvettes with 2 mm flight path as described in^9^. Background correction was performed for all data sets using scattering data obtained with pure *D*_2_*O*. Scattered intensity *I*(*q*) is measured as a function of the scattering vector *q* = 4*πsin*(2*θ*)∕*λ*, where *λ* is the wavelength and 2*θ* is the scattering angle. Typical Δ*q*/*q* values were between 10 and 20% and the sample-to-detector distance was 10.55 m. Instrumental resolution was taken into account during data treatment. The vanadium standard was used to express *I*(*q*) in absolute units. During the measurements, the temperature of the samples was controlled by a water thermostat with an accuracy of  ± 0.05 °C^70^.

### Neutron spin echo experiments

NSE experiments were performed using the MESS (LLB, Saclay) instrument. Data were collected at the wavelength *λ* = 6 Å and *q* = 0.1096 Å^−1^ that corresponds to the scattering angle 2*θ* = 6°. Experiments were performed in the temperature and Fourier time ranges of 20–32 °C and 0–17 ns. Each lipid sample was transferred in a quartz cuvette with 4 mm flight path. Background corrections were done for all data sets using 4% *H*_2_*O* in *D*_2_*O* (v/v). The samples were measured two times using a stepwise increase in the temperature 0.1–0.2 °C. The sample temperature was controlled with an accuracy of  ± 0.05 °C.

## Supplementary information

Supplemental material.
