# Supplementary material for: On the Origin of the Anomalous Behavior of Lipid Membrane Properties in the Vicinity of the Chain-Melting Phase Transition
Source: Sci Rep. 2020 Apr 1;10:5749. doi: 10.1038/s41598-020-62577-9 (PMC7113312; doi:10.1038/s41598-020-62577-9)
Supplement: Supplementary file 1 — Supplemental material. [file 41598_2020_62577_MOESM1_ESM.pdf]

# Supplemental material

On the Origin of the Anomalous Behavior of Lipid Membrane Properties in the Vicinity of the Chain-Melting Phase Transition

A. Kuklin, D. Zabelskii, I. Gordeliy, J. Teixeira, A. Brûlet, V. Chupin, V. Cherezov and V. Gordeliy

December 16, 2019

## SANS data

Representative examples of the SANS diffraction data of 4% (v/v) DMPC MLVs in $D_2O$ are shown in Figure S1A for $T = 18\,°C, 23\,°C$ and $24\,°C$. The data are presented in $Iq^2$ vs $q$ coordinates (Kratky-Porod plot) after normalization and background subtraction. Multiplication of the scattering intensity $I(q)$ by the Lorentz factor $q^2$ was done to correct for the scattering from randomly-oriented multilayers. The first order diffraction peak around $q = 0.1Å^{-1}$ is clearly visible at all temperatures, and an additional peak around $q = 0.05Å^{-1}$, although weak, is visible at $T = 18\,°C$. It corresponds to a ripple phase of DMPC with the periodicity of 120Å. The repeat distances, $d$, of multilayers were calculated using the Bragg equation: $2d \sin\theta = n\lambda$ [1].

For SANS experiments with 1% (v/v) DMPC ULVs in $D_2O$ selected scattering curves are shown for $T = 18$ and $25\,°C$ (Figure S1B). The results are plotted using the Kratky-Porod plot in log-lin representation according to the asymptotic expression for the scattering from lipid bilayers [2]:

$$I(q) = \frac{I_0}{q^2} exp(-R_T^2 q^2) \tag{S1}$$

which can be applied when $1/\sqrt{S} < q < 1/R_T$ where $S$ is the vesicle surface area and $R_T$ is the bilayer radius of gyration which is given by:

$$R_T^2 = \frac{\int_S \rho(z) z^2 dz}{\int_S \rho(z) dz} \tag{S2}$$

Calculations of the bilayer thickness using $R_T$ were done by using $d_b = R_T\sqrt{12}$ with subsequent correction for the hydration of polar lipid heads and inhomogeneity of the scattering [3]:

$$d_b' = d_b\left(1 - 1.26\frac{V_W}{V_L}\right) \tag{S3}$$

where $V_W = n_W \nu_{WL} = 7.2 \cdot 30Å^3 = 216Å^3$ is the volume of water molecules inside the bilayer and $V_L$ is the volume of a DMPC molecule ($V_L = 1101Å^3$ [4])

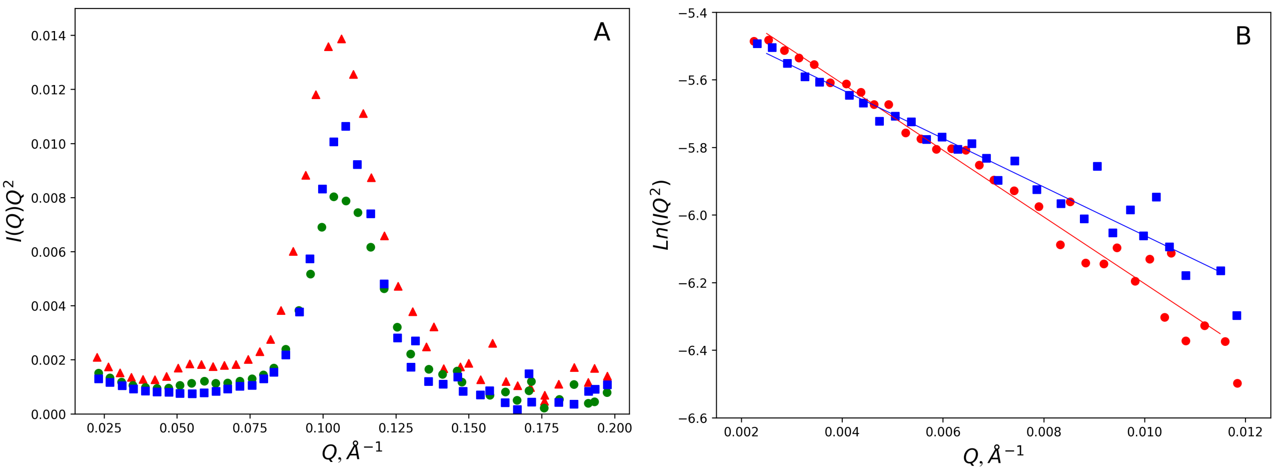


Figure S1: (A) Representative examples of diffraction data for 4% (v/v) DMPC MLVs in $D_2O$ presented in $Iq^2$ vs $q^2$ plot at $T = 18\,°C, 23\,°C$ and $24\,°C$ and colored in red, green and blue, respectively. (B) Examples of SANS data for 1% (v/v) DMPC ULVs in $D_2O$ presented in $ln(Iq^2)$ vs $q^2$ plot at $T = 18\,°C$ and $25\,°C$ and colored in red and blue, respectively

## NSE spectra fitting

Spin echo signal from undulating vesicles consist of two different parts: Static form-factor of a vesicle, which corresponds to the contribution of the diffusion term and quasielastic form-factor which accounts for undulations of the vesicle:

$$I(q,t,r) = e^{-Dq^2t} V_s^2 (\Delta\rho)^2 I_1(q,t,r) \tag{S4}$$

where $I_1$ is given by:

$$I_1(q,t,r) = \Big( f_0(qr) + \sum_{l=1} \frac{2l+1}{4\pi} \big\langle a_1(0) a_1(t) \big\rangle \Big) \tag{S5}$$

$f_0 = (sin(qr)/qr)^2$ is the static form factor, $f_1 = \big((l+2)j_l(qr) - qrj_{l+1}(qr)\big)^2$ is the quasielastic form-factor, $r$ is the radius of a vesicle, $V_s$ is its scattering volume, $\Delta\rho$ is the scattering density contrast, $j_l$ is the $l_{th}$ Bessel function, $D = kT/6\pi\eta r^*$ is the diffusion coefficient, where $r^* = r + 25\text{Å}$ is the hydrodynamic radius, $\eta$ is the $D_2O$ viscosity, $k_B$ is the Boltzmann constant and $\big\langle a_1(0)a_1(t)\big\rangle = \langle a_l^2 \rangle exp(-\omega_l t)$ is the autocorrelation function of the undulation amplitude, where the average undulation amplitude is given by:

$$\langle |a_l^2| \rangle = \frac{k_B T}{K_c} \frac{l^2(l+1)^2(l+2)(l-1)}{(2l+1)(2l^2+2l+1)} \tag{S6}$$

$K_c$ is the bending rigidity and $l$ is the undulation mode. The undulation frequency is given by the expression:

$$\omega_l = \frac{K_c}{\eta r^3} \frac{l^2(l+1)^2(l+2)(l-1)}{(2l+1)(2l^2+2l+1)} \tag{S7}$$

The polydispersity of vesicles is taken into account by a Gaussian distribution:

$$p(r) = \sqrt{\frac{1}{2\pi\sigma^2}} exp\Big( - \frac{(r-R_0)^2}{2\sigma^2} \Big) \tag{S8}$$

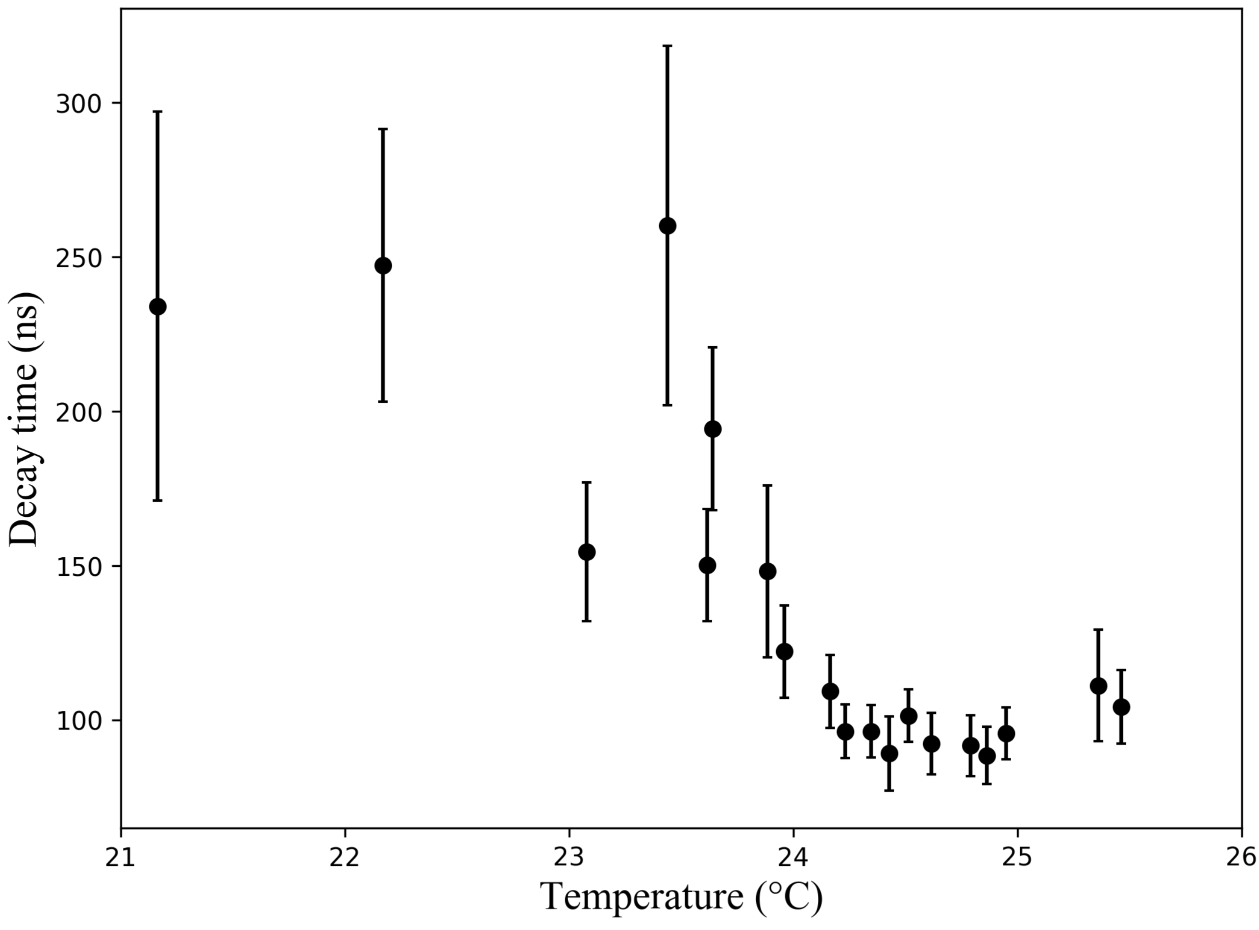


Figure S2: Temperature dependence of the decay time for 4% (v/v) DMPC ULVs in $D_2O$ obtained with single exponential fits of the NSE data near the chain-melting phase transition region.

The scattering intensity from polydisperse vesicles can be written as:

$$I(q,t) = \int_{r_{min}}^{r_{max}} I(q,t,r)p(r)dr \tag{S9}$$

For vesicles made with a small volume extrusion apparatus, the polydispersity $\sigma_R \approx 0.3R_0$, as it was shown previously in [5]. For diffusion motion of rigid vesicles, the NSE decay time is given by:

$$\tau = \frac{1}{Dq^2} = \frac{6\pi\eta r^*}{kTq^2} \tag{S10}$$

The NSE data can be ambiguously treated with any number of exponents in quasielastic form-factor, although NSE signal can be easily overfitted. The decay times of the MLVs obtained from single exponential fits of the data are presented at Figure S2. The data clearly demonstrate that decay time dependence decreases by factor of 3, when it should nearly the same, that indicate the strong contribution from the undulation term which should be taken into account during data processing.

# References


[1] V. Gordeliy and M. Kiselev, "Definition of lipid membrane structural parameters from neutronographic experiments with the help of the strip function model," *Biophysical journal*, vol. 69, no. 4, pp. 1424–1428, 1995.

[2] W. Knoll, K. Ibel, and E. Sackmann, "Small-angle neutron scattering study of lipid phase diagrams by the contrast variation method," *Biochemistry*, vol. 20, no. 22, pp. 6379–6383, 1981.

[3] V. Gordeliy, L. Golubchikova, A. Kuklin, A. Syrykh, and A. Watts, "The study of single biological and model membranes via small-angle neutron scattering," in *Trends in Colloid and Interface Science VII*, pp. 252–256, Springer, 1993.

[4] J. F. Nagle and S. Tristram-Nagle, "Structure of lipid bilayers," *Biochimica et Biophysica Acta (BBA)-Reviews on Biomembranes*, vol. 1469, no. 3, pp. 159–195, 2000.

[5] R. C. MacDonald, R. I. MacDonald, B. P. M. Menco, K. Takeshita, N. K. Subbarao, and L.-r. Hu, "Small-volume extrusion apparatus for preparation of large, unilamellar vesicles," *Biochimica et Biophysica Acta (BBA)-Biomembranes*, vol. 1061, no. 2, pp. 297–303, 1991.
